# A Lombard Variety of Sweet Pepper Regulating Senescence and Proliferation: The Voghera Pepper

**DOI:** 10.3390/nu16111681

**Published:** 2024-05-29

**Authors:** Fabrizio De Luca, Federica Gola, Alberto Azzalin, Claudio Casali, Ludovica Gaiaschi, Gloria Milanesi, Riccardo Vicini, Paola Rossi, Maria Grazia Bottone

**Affiliations:** 1 Department of Biology and Biotechnology, University of Pavia, 27100 Pavia, Italy; federica.gola01@universitadipavia.it (F.G.); alberto.azzalin@unipv.it (A.A.); claudio.casali@unipv.it (C.C.); ludovica.gaiaschi@unipv.it (L.G.); gloria.milanesi@unipv.it (G.M.); paola.rossi@unipv.it (P.R.); mariagrazia.bottone@unipv.it (M.G.B.); 2 Bio Basic Europe S.R.L., 27100 Pavia, Italy; riccardo.vicini@biobasiceurope.it

**Keywords:** phytotherapy, nutraceuticals, Voghera sweet pepper, ascorbic acid, normal human dermal fibroblast, aging, senescence, cell proliferation

## Abstract

Aging and its related disorders are important issues nowadays and the first cause of this physio-pathological condition is the overproduction of ROS. Ascorbic acid is an antioxidant mediator and its anti-aging proprieties are well known. Our previous data demonstrated that Voghera sweet pepper (VP), a distinctive type of pepper cultivated in Italy, is particularly rich in ascorbic acid. Based on these data, the anti-aging effect mediated by extracts of the edible part of VP was evaluated on an in vitro model of both young and old Normal Human Diploid Fibroblasts (NHDF). Using phase contrast microscopy, we observed that VP may help cells in the maintenance of physiological morphology during aging. Cytofluorimetric analyses revealed that VP extracts led to an increase in DNA synthesis and percentage of living cells, linked to a consequent increase in mitotic events. This hypothesis is supported by the enhancement of PCNA expression levels observed in old, treated fibroblasts, corroborating the idea that this extract could recover a young phenotype in adult fibroblasts, confirmed by the study of p16 and p53 expression levels and TEM analyses. Based on these results, we may suppose that VP can lead to the partial recovery of “young-like” phenotypes in old fibroblasts.

## 1. Introduction

Aging is a biological process characterized by the progressive decline of physiological functions that involve the entire organism, from individual cells to organs [1,2]. Overproduction of reactive oxygen species (ROS) and the consequent increase in oxidative stress levels are among the main causes of aging and its related lifelong pathological conditions such as hypertension, cancers, diabetes, atherosclerosis, Alzheimer’s disease, and Parkinson’s disorder [3,4]. The excess of free radicals rapidly leads to the activation of the inflammatory pathway and subsequent protein oxidation, lipid peroxidation, mitochondrial, and DNA damage [5]. From a molecular standpoint, cellular senescence is characterized by morphological changes, such as enlarged and flattened cell morphology, high activity of senescence-associated beta-galactosidase (SA-β-gal), increases in senescence-associated secretory phenotype (SASP) characterized by cytokine and matrix metalloproteinase expression, a reduction in lamin B1 expression, cytoplasmic translocation of nuclear HMGB1 senescence-associated β-galactosidase (β-Gal) activity, as well as overexpression of cell cycle arrest proteins (e.g., p16 and p53) [6], and a decrease in the expression levels of specific markers of cell proliferation (e.g., proliferating cell nuclear antigen (PCNA)) [7,8,9].

In recent years, more attention has been paid to various natural compounds such as carotenoids, tocopherols, and flavonoids, as well as vitamins (A, C, D, and E) due to their antioxidant properties and potentially beneficial biological activities for inhibiting or treating aging [10,11]. In particular, ascorbic acid is a natural molecule with confirmed antimicrobial, immunomodulatory, anti-inflammatory, and antioxidant properties that have been known since the 1930s. However, unlike the vast majority of vertebrates, humans are not able to synthesize this vitamin and, therefore, need to acquire ascorbic acid from the diet [12,13,14]. Additionally, oxidative stress is the most significant cause of aging, and the anti-inflammatory and antioxidant properties of vitamin C, a well-known free radical scavenger, have been extensively reported in the literature [4,15].

In this regard, sweet peppers (*Capsicum annuum*), one of the most important vegetables in the world due to their high nutritional value, are very rich in bioactive compounds such as polyphenols, β-carotene, and vitamin C, with the amount of biological molecules varying based on the type of pepper considered and its degree of maturity [16,17].

In Italy, diverse climatic and cultivation conditions have favored the selection of a wide variety of pepper cultivars and local landraces, which represent a valuable, underexploited resource [18].

The Voghera pepper, a native Lombardy variety of vegetable cultivated in Italy between the provinces of Alessandria and Pavia by a limited number of producers as a niche product, disappeared from our diet in the past century and was subsequently reintroduced and cultivated again in 2006 [19]. The high nutritional value of this variety of pepper, rich in vitamin C and carotenoids, and its role in protection against oxidative damage have been previously reported in the literature, suggesting the importance that these pepper varieties may have as a potential anti-aging vegetable in the diet [20].

Based on previous literature data regarding the antioxidant properties of Voghera pepper, our research aimed to evaluate the possible anti-aging effect mediated by the high levels of vitamin C detected in extracts of the edible part of Voghera pepper, comparing them to another important northern Italian variety, i.e., Carmagnola pepper (CP), whose beneficial properties have already been reported [18].

In particular, we investigated cell morphology, ultrastructural and cell cycle changes, and the probable modifications in the expression levels of specific markers of proliferation and aging using an in vitro model of both young and aged Normal Human Diploid Fibroblasts (NHDF).

## 2. Materials and Methods

### 2.1. Cell Cultures and Treatments 

Normal Human Diploid Fibroblasts (NHDF), generously provided by Bio Basic S.r.l. (Pavia, Italy), were cultured in Dulbecco’s Modified Eagle Medium (DMEM) supplemented with 10% fetal bovine serum and penicillin/streptomycin (100 IU/50 µg/mL), and maintained in a humidified atmosphere containing 5% CO_2_ in air at 37 °C. Young NHDF at passage 18 were utilized for the experiments. Replicative senescent NHDF were induced by long-term passaging of the cells in cell culture. Cells acquired the senescence phenotype at passage 48 [20]. Forty-eight hours before the experiments, NHDF were seeded: (i) for phase contrast microscopy and fluorescence analyses (200,000 cells/glass coverslip) and (ii) for cell cycle and TEM evaluations (1 × 10^6^ cells). Young and old fibroblasts were treated for 24 h with Voghera pepper (VP) and control Carmagnola pepper (CP) extracts at a concentration of 1 mg/mL. This concentration was determined based on the results of the MTT assay performed in our previous study [20], as well as previous literature data concerning other natural extracts [21,22].

Regarding extract preparation (Table 1) and characterization, peppers were extracted in a mixture of water and 1,2-propanediol (45% water–55% 1,2-propanediol) and subsequently analyzed using a HPLC–UV–Vis system. For an extensive description of the extraction procedure and chemical composition of the studied extracts, refer to reference [19].

### 2.2. Clonal Cell Survival Assay

Cells were plated in a 6-well plate at a density of 500 cells/well in 1000 µL of proliferation medium (PM) containing different pepper extracts (i.e., Voghera or Carmagnola) and incubated for 24 h. Subsequently, the treatment medium was removed, fresh medium was added, and cells were further incubated for 9 days. The nuclei were then counterstained using hematoxylin, and colonies were counted. For each experimental condition, six independent experiments were carried out. Plating efficiency-based (PE) calculation of survival fractions was performed as previously reported [23]. Briefly, PE values were determined by dividing the number of colonies obtained by the number of cells seeded under untreated conditions, and survival fractions (SFs) were calculated by dividing the number of colonies obtained by the number of cells seeded per pepper extract, multiplied by PE.

After counting clones, plating efficiency (PE) and survival fraction (SF) were calculated using the following formulas:

PE% = # of colonies formed/# of cells seeded × 100%

SF = # of colonies formed after treatment/(# of cells seeded × PE)

### 2.3. Phase Contrast Microscopy

NHDF cells were observed under inverted phase contrast microscopy equipped with a ×20 or ×40 objective (Olympus CKX41, Evident Europe GmbH, Hamburg, Germany) after treatment for 24 h to evaluate possible alterations in cell morphology in young and old fibroblasts. Digital pictures were acquired with a camera (Olympus MagniFire digital camera, Evident Europe GmbH, Hamburg, Germany) and processed using Olympus Cell F software (version 3.1).

Cell morphology analysis was performed using ImageJ software 1.51 (NIH, Wellesley, MA, USA, version 1.54f). Specifically, five random images, for each experimental condition, were captured in each well of 6-well culture plates (excluding areas near the edge of the well) at 40× magnification. Subsequently, the images were converted to binary and analyzed using the “particle analysis tool”. The area of 10 cells, expressed in µm^2^, was calculated for each image. Data are presented as the mean ± SEM. For each experimental condition, six independent experiments were carried out.

### 2.4. Flow Cytometry, Cell Cycle and Cell Death Analyses

Flow cytometry analyses in control and treated NHDF cells were conducted as previously described [24]. Specifically, following a 24 h treatment, NHDF cells were detached through mild trypsinization (0.25% in PBS, with 0.05% EDTA) to obtain single-cell suspensions for flow cytometry processing using a Partec Cy-Flow Space system (Sysmex-Partec, Milan, Italy), equipped with an argon ion laser excitation at 488 nm (power 200 mW). Data were analyzed using the built-in software (Flowmax, Partec, version 2.70).

A subset of NHDF samples was washed in PBS, fixed in cold 70% ethanol (−20 °C) for at least 60 min, and refrigerated (+4 °C) until all samples were collected. Subsequently, the samples were processed for cell cycle assessment by flow cytometry: fixed cells were washed in PBS to remove ethanol and resuspended in PBS containing RNase A (100 U/mL), Nonidet P 40 (0.1%) (Sigma-Aldrich, Milan, Italy), and propidium iodide (PI) (50 µg/mL) (Sigma-Aldrich, Milan, Italy). The cells were kept in this staining solution for at least 24 h before flow analysis. Flow cytometry analyses were performed using a Partec Cy-Flow Space system (Sysmex-Partec, Milan, Italy), equipped with an argon ion laser excitation at 488 nm (power 200 mW), and data were analyzed using the built-in software (Flowmax, Partec, version 2.70).

Another subset of NHDF samples was processed for the identification of living cells. Briefly, NHDF cells were quickly washed in PBS, permeabilized in 70% ethanol for 10 min, treated with RNase A (100 U/mL), and then stained at room temperature with propidium iodide (PI) (50 μg/mL) (Sigma-Aldrich, Milan, Italy) 1 h before flow cytometric analysis. For both analyses, PI red fluorescence was detected with a 610 nm long-pass emission filter. At least 10,000 cells per sample were measured to obtain the distribution among the different phases of the cell cycle and to assess the percentage of living cells normalized to the control. For each experimental condition, three independent experiments were carried out. 

### 2.5. Immunofluorescence Reactions

Control and treated NHDF cells were fixed with 4% formalin for 20 min and post-fixed with 70% ethanol at −20 °C for at least 24 h until staining. The samples were rehydrated for 10 min in PBS-Tween 0.2%, and then nonspecific sites were blocked using a blocking solution of PBS supplemented with 2% BSA and 0.2% Tween for 15 min at room temperature (RT). Subsequently, cells were immunolabeled with primary antibodies (Table 2) diluted in PBS-Tween 0.2% for 1 h at RT in a moist chamber. After washing in PBS, coverslips were incubated with secondary antibodies (Table 2) in PBS-Tween 0.2% for 30 min. At the end of the incubation, after washing in PBS, cells were counterstained for DNA with 0.1 µg/mL Hoechst 33258; then, cells were washed with PBS and finally mounted in a drop of Mowiol (Calbiochem-Inalco S.r.l., Milan, Italy) for fluorescent microscopy. For each experimental condition, three independent experiments were carried out.

### 2.6. Fluorescence Microscopy

An Olympus BX51 microscope (Evident Europe GmbH, Hamburg, Germany) equipped with a 100 W mercury lamp was used under the following conditions: 330–385 nm excitation filter (excf), 400 nm dichroic mirror (dm), and 420 nm barrier filter (bf) for Hoechst 33258; 450–480 nm excitation filter (excf), 500 nm dm, and 515 nm bf for the fluorescence of Alexa 488; 540 nm excitation filter (excf), 580 nm dm, and 620 nm bf for Alexa 594. Images were recorded with an Olympus MagniFire camera system and processed using the Olympus Cell F software (version 3.1).

### 2.7. Immunofluorescence Analyses

After immunocytochemical reactions, images were recorded using Cell F software. The time exposure during acquisition was determined on the control sample and then maintained constant for all experimental conditions to ensure comparability of fluorescence intensity between different experimental conditions. Subsequently, the fluorescence intensity was analyzed using ImageJ software. 

### 2.8. Transmission Electron Microscopy (TEM) 

Control and treated cells were processed for TEM analysis as described below. Briefly, cells were harvested by mild trypsinization (0.25% trypsin in PBS containing 0.05% EDTA) and centrifuged at 800 rpm for 10 min. The samples were immediately fixed with 2.5% glutaraldehyde (Polysciences, Inc., Warrington, PA, USA) in culture medium (2 h at room temperature) and washed three times with PBS (10 min each). Samples were then stained in 1% OsO4 (Sigma Chemical Co., St. Louis, MO, USA) for 2 h at room temperature and washed in distilled water. The cell pellets were pre-embedded in 2% agar, dehydrated with increasing concentrations of acetone, and finally embedded in epoxy resin. Ultrathin sections (70–80 nm) were cut on a Reichert OM-U3 ultramicrotome (Reichert OM-U3 ultramicrotome, Leica, Wetzlar, Germany), collected on nickel grids, and stained with lead citrate. Lastly, sections were observed under a JEM 1200 EX II (JEOL, Peabody, MA, USA) electron microscope, equipped with a MegaView G2 CCD camera (Olympus OSIS, Tokyo, Japan), operating at 100 kV, and processed using the iTEM software (version 5.1).

### 2.9. Statistical Analysis

Data are presented as the mean ± SEM. The Anderson–Darling, D’Agostino and Pearson, Shapiro–Wilk, and Kolmogorov–Smirnov tests were used to assess the normality of parameters. Subsequently, data were analyzed to determine statistically significant differences. For data that passed the normality test, analysis was conducted using one-way ANOVA followed by Bonferroni’s post hoc test for multiple comparisons. Conversely, for non-normally distributed results, analysis was performed using the Kruskal–Wallis test followed by Dunn’s test. Statistical significance was considered as *p* < 0.05 (*), *p* < 0.01 (**), and *p* < 0.001 (***). All statistical analyses were performed using GraphPad Prism 8.0 (GraphPad Software Inc., CA, USA).

## 3. Results

**Figure 1:**
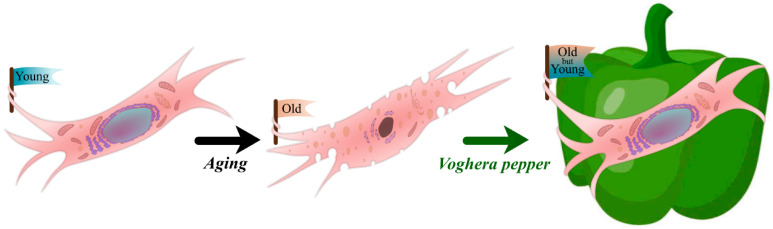
Graphic illustration underlining the ability of Voghera pepper in the recovery of young phenotype in aged fibroblasts.

In the present study, Normal Human Diploid Fibroblasts (NHDF) were chosen to evaluate the anti-aging effect of Voghera pepper extracts and their capacity to regulate cell cycle and proliferation, as well as their ability to modify cellular ultrastructure in both young and senescent fibroblasts, compared to control pepper extract, i.e., Carmagnola pepper. Our goal was to understand the key mechanisms behind the anti-aging effect of this bell pepper variety (Figure 1). A specific concentration of Voghera and Carmagnola pepper extracts was used, based on our previous study [20]. 

### 3.1. Effects on Cell Morphology

**Figure 2:**
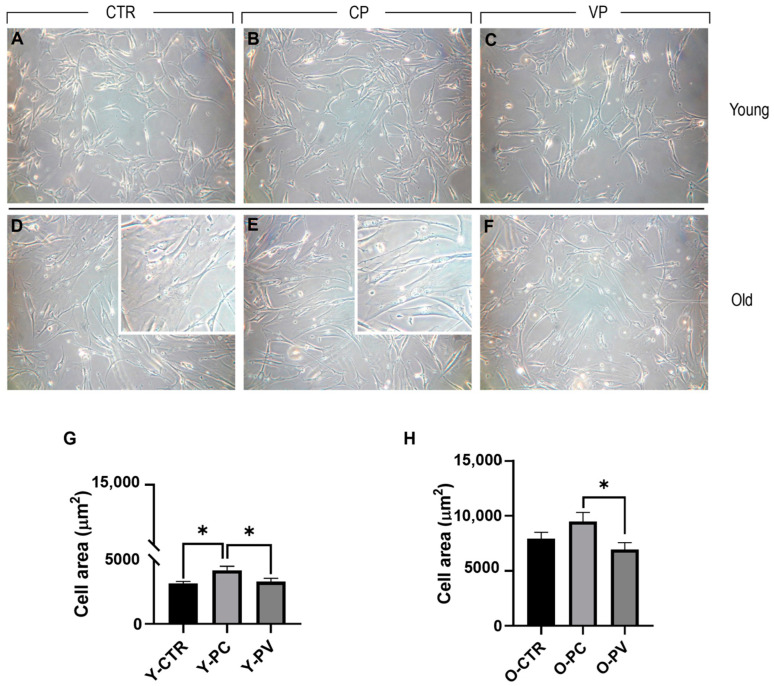
Phase contrast microscopy of young (**A**–**C**) and old (**D**–**F**) NHDF treated differently, i.e., CTR (**A**,**D**), CP (**B**,**E**), and VP (**C**,**F**), respectively. Histograms illustrating the quantitative cell area analysis in young (**G**) and old (**H**) NHDF. *p* values calculated by one-way ANOVA followed by Bonferroni’s post hoc test: (*) <0.05. Magnification: 20× (**A**–**F**); 40× (insert in **D**,**E**). Data are expressed as the mean ± SEM.

After 24 h exposure to Voghera or Carmagnola pepper extracts, young and old NHDF were plated on 1 cm^2^ round coverslips and examined. In detail, phase contrast microscopy (Figure 2) evaluation revealed the absence of any morphological alteration in both young and old control NDHF, as well as after Voghera or Carmagnola pepper treatment, with fibroblasts showing the typical elongated shape. However, we observed the presence of several cells distributed in old groups that displayed an extremely flattened and enlarged soma, compared to other cells, detectable in all old experimental groups but more evident in ctr and CP NHDF (insert in D and E, respectively). Regarding young NHDF, a significant increase in cell area was highlighted in CP, compared to both control and VP groups. No significant difference was observed comparing control and VP NHDF (Panel G). Conversely, old control NHDF cells displayed an enlarged soma, with almost double the cell area compared to young control fibroblasts, and a further slight increase in cellular surface was observed after CP treatment. However, a slight decrease in cell area was detected in VP NHDF cells compared to control. However, this reduction in cell area became significant when comparing VP and CP experimental groups (Panel H) (Table 3). 

### 3.2. Pepper Extracts Differently Affect Young and Old NHDF Clonogenic Capability

**Figure 3:**
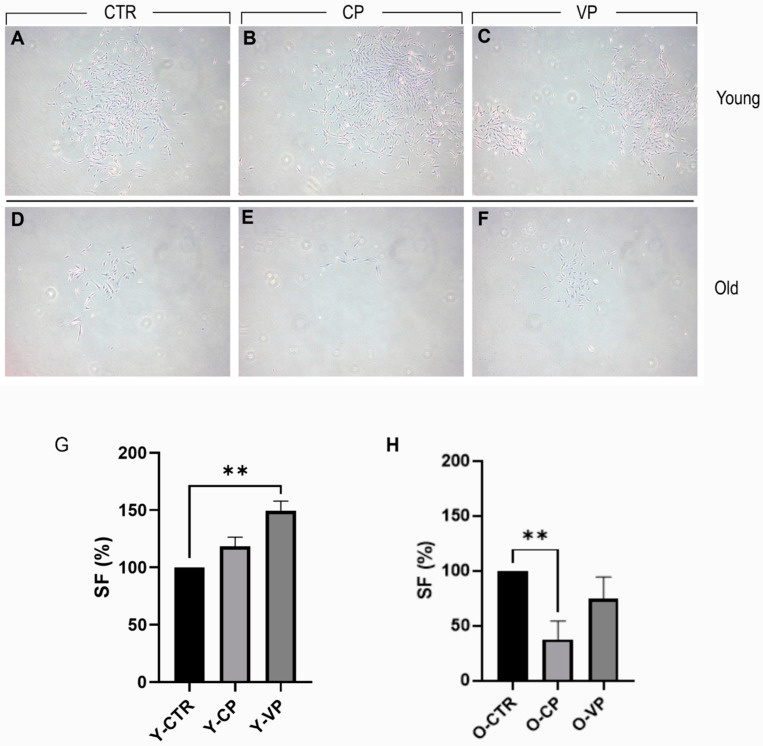
Clonogenic cell survival assay showing the treatment–response effect of CTR (**A**,**D**), CP (**B**,**E**) and VP (**C**,**F**) treatment, in both young and old NHDF (**A**–**C** and **D**–**F**, respectively) after 9 days of exposure. Histogram showing the young (**G**) and old (**H**) NHDF survival fraction (SF%) in control, CP and VP. *p* values calculated by Kruskal–Wallis test followed by Dunn’s test. (**) <0.01. Magnification: 10× (**A**–**F**). Data are expressed as the mean ± SEM.

For the clonal analysis, Figure 3 presents a typical result of the 9-day clonogenic assay. We observed a slight increase in the clonogenic capability of young NHDF (survival fraction [SF] approximately 118%) after CP treatment, with a further statistically significant improvement after VP exposure (SF approximately 150%). Conversely, a significant reduction in the clonogenic capability of old NHDF (SF approximately 38%) was observed in the CP-treated group. However, only a slight decrease was reported after VP treatment (SF approximately 75%) (Table 4).

### 3.3. Cell Cycle Distribution and Cell Death

**Figure 4:**
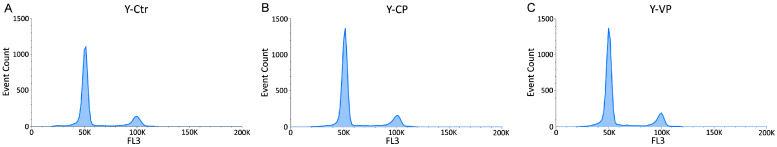
Flow cytometry data reporting cell cycle NHDF analysis. Histograms showing the DNA content after propidium iodide (PI) staining in young (Y) NHDF Dual parameter cytograms of event count and PI staining (FL3) in controls (**A**), CP- (**B**) and VP- (**C**) treated cells.

**Figure 5:**
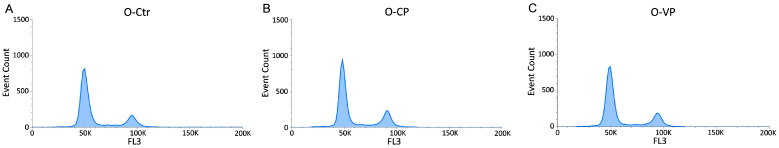
Flow cytometry data reporting cell cycle NHDF analysis. Histograms showing the DNA content after propidium iodide (PI) staining in old NHDF. Dual parameter cytograms of event count and PI staining (FL3) in controls (**A**), CP- (**B**) and VP- (**C**) treated cells.

The cytofluorimetric analysis in young and old groups revealed that, similarly to the control, both young and old NHDF treated with CP or VP were normally scattered among the different cell phases (G1, S, and G2), showing a physiological DNA content distribution. However, a slight non-statistically significant (*p* > 0.05) increase in the S phase was observed in both young and old CP NHDF (about 4.03 ± 1.56% and 4.13 ± 1.33% for Y-CP and O-CP, respectively), with a further insignificant (*p* > 0.05) increase after VP exposure (about 4.98 ± 1.88% and 6.32 ± 2.5% for Y-VP and O-VP, respectively) (Figure 4 and Figure 5).

**Figure 6:**
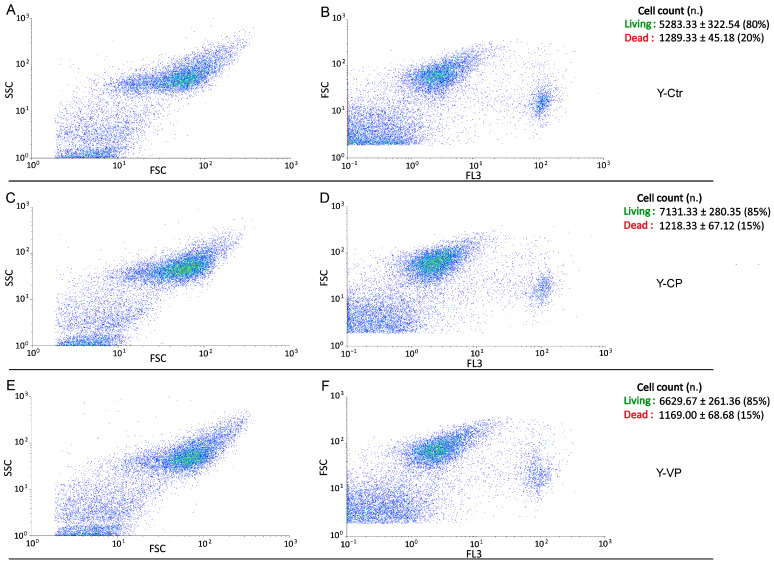
Flow cytometry data reporting live/death young NHDF analysis immediately after CP or VP exposure. Cytograms showing the number of live/death cells after propidium iodide (PI) staining in young NHDF. Dual parameter cytograms of side scatter (SSC) vs. forward scatter (FSC) and forward scatter (FSC) vs. PI staining (FL3) in controls (**A**,**B**), CP- (**C**,**D**) and VP- (**E**,**F**) treated cells. Data are expressed as the mean ± SEM.

**Figure 7:**
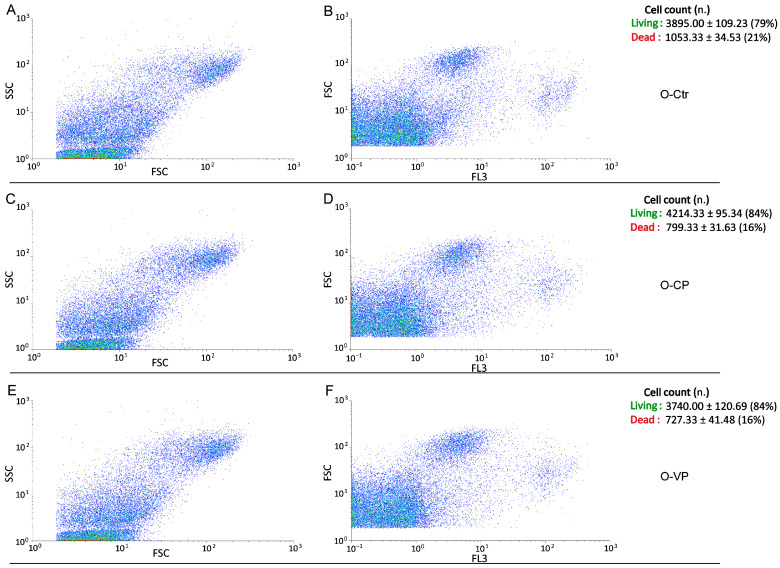
Flow cytometry data reporting live/death old NHDF analysis immediately after CP or VP exposure. Cytograms showing the number of live/death cells after propidium iodide (PI) staining in old NHDF. Dual parameter cytograms of side scatter (SSC) vs. forward scatter (FSC) and forward scatter (FSC) vs. PI staining (FL3) in controls (**A**,**B**), CP- (**C**,**D**) and VP- (**E**,**F**) treated cells. Data are expressed as the mean ± SEM.

Conversely, regarding the assessment of cell death, the evaluation of bi-parametric cytograms revealed that both CP and VP extracts were able to significantly (*p* < 0.05 (*)) increase the number of living cells (about 5% and 5%, respectively) after 24 h exposure in young NHDF (Figure 6), with a similar but not significant (*p* > 0.05) increase observed in the old experimental groups (about 5% and 5%, respectively) (Figure 7). 

### 3.4. Cellular Proliferation

**Figure 8:**
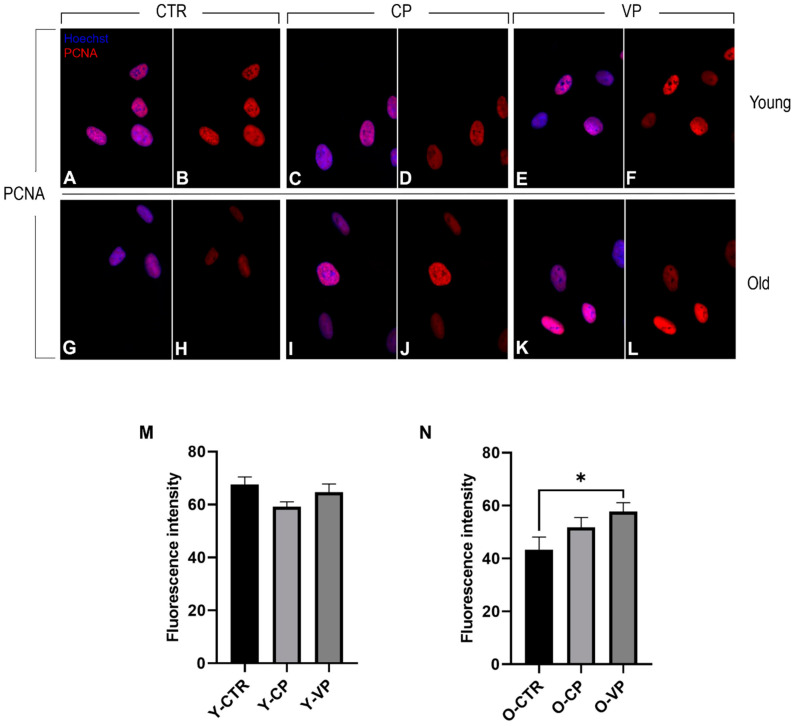
Immunocytochemical detection of PCNA (red signal) by fluorescence microscopy in CTR (**A**,**B**,**G**,**H**), CP (**C**,**D**,**I**,**J**) and VP (**E**,**F**,**K**,**L**) treatment, in both young and old NHDF (**A**–**F** and **G**–**L**, respectively). DNA counterstaining with Hoechst 33258 (blue fluorescence). Histograms depicting the quantitative measurement of young (panel **M**) and old (panel **N**) PCNA mean fluorescence intensity per cell. Statistically significant data: * *p* < 0.05. Magnification: 60×. Data are expressed as the mean ± SEM.

The immunoreaction for PCNA (red fluorescence) revealed strong localized and confined PCNA immunopositivity in the nucleus of both young and old NHDF treated differently. In particular, no statistically significant differences in PCNA immunopositivity were observed among the young experimental groups, although a slight decrease in PCNA labeling was detected when comparing the control group with both the CP- and the VP-treated cells (Figure 8). Conversely, PCNA immunolabeling was significantly increased when comparing old control cells with differently aged treated fibroblasts. Specifically, a slight increase in immunopositivity was detected in O-CP cells compared to O-CTR (*p* = 0.42). Notably, a significant enhancement of PCNA immunoreaction was observed when comparing O-VP and O-CTR NHDF (*p* = 0.04) (Figure 8) (Table 5).

### 3.5. Anti-Aging Effects

The anti-aging activity of pepper extracts was evaluated through the analysis of the expression levels of two different senescence markers: p53 and p16. 

**Figure 9:**
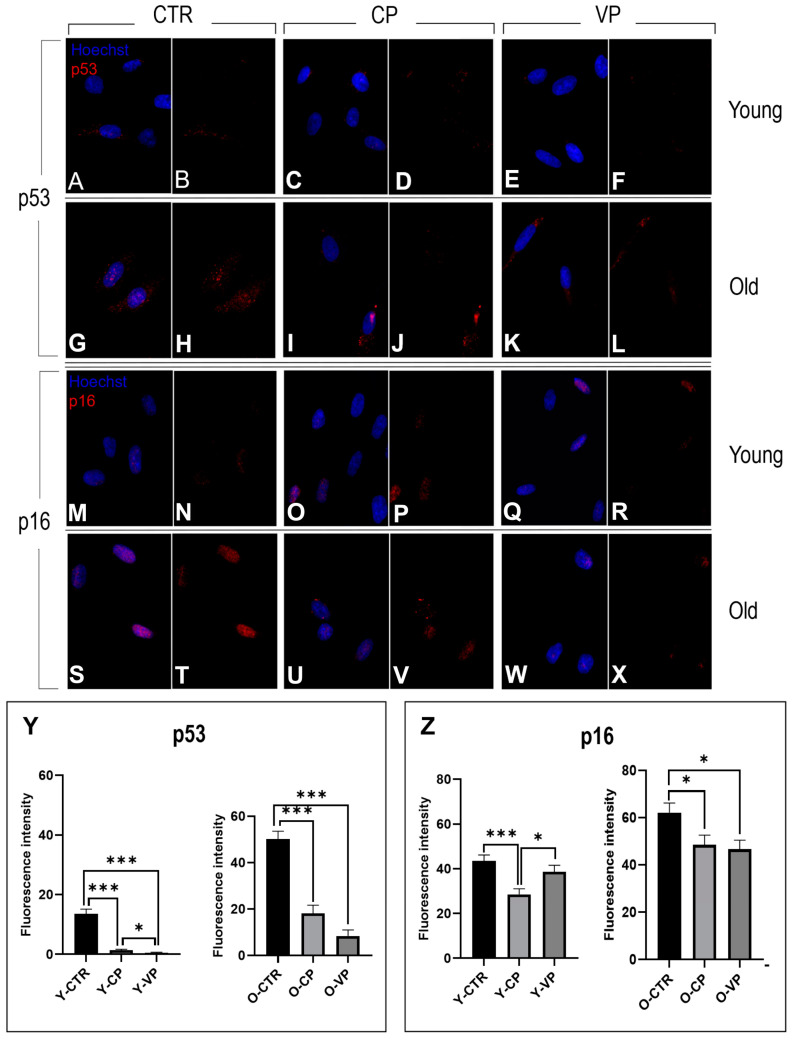
Immunocytochemical detection of p53 (red signal) and p16 (red signal) by fluorescence microscopy in CTR (**A**,**B**,**G**,**H** and **M**,**N**,**S**,**T** for p53 and p16, respectively), CP (**C**,**D**,**I**,**J** and **O**,**P**,**U**,**V** for p53 and p16, respectively) and VP (**E**,**F**,**K**,**L** and **Q**,**R**,**W**,**X** for p53 and p16, respectively) treatment, in both young and old NHDF (**A**–**F**, **M**–**R** and **G**–**L**, **S**–**X**, respectively). DNA counterstaining with Hoechst 33258 (blue fluorescence). Histograms depicting the quantitative measurement of p53 (Panel **Y**) and p16 (Panel **Z**) mean fluorescence intensity per cell, in young and old NHDF (left and right histograms, respectively). Statistically significant data: * *p* < 0.05, *** *p* < 0.001. Magnification: 60×. Data are expressed as the mean ± SEM.

Immunocytochemical reactions for p53 and p16 revealed well-defined immuno-positivity in the nucleus of both young and old NHDF treated differently. Slight immuno-labeling of p16 and p53 was also observed in the cytoplasm of several cells, with no differences among the experimental groups). In particular, regarding the expression levels of p53 (red fluorescence), we observed a significant decrease in young NHDF when comparing the control group with the differently treated NHDF (Y-CTR vs. Y-CP: *p* < 0.001; Y-CTR vs. VP: *p* < 0.001). Notably, a significant reduction was also observed between young VP and CP cells (*p* = 0.02) (Figure 9, Panel Y, left). Similarly to the young groups, the old differently treated cells displayed a significant decrease in p53 immunopositivity compared to O-CTRL NHDF (O-CTR vs. CP: *p* < 0.001; Y-CTR vs. VP: *p* < 0.001). A slight decrease in p53 immunopositivity was detected between O-VP and O-CP NHDF (Figure 9, Panel Y, right) (Table 6). 

Additionally, the p16 immunoreaction showed a decrease in fluorescence intensity in the young differently treated groups compared to young untreated cells, with an extremely significant decrease when comparing Y-CP and Y-CTR (*p* = 0.0005) and only a significant decrease compared to Y-VP (Figure 9, Panel Z, left).

Finally, in the old groups, our investigations revealed that p16 exhibited a decreasing trend when comparing the control with the treated cells; in particular, p16 expression was significantly reduced in the fibroblasts treated with both CP (*p* = 0.05) and Voghera pepper extracts (*p* = 0.02) (Figure 9, Panel Z, right) (Table 7).

### 3.6. TEM

Transmission electron microscopy highlighted the presence of a well-organized nucleus in young control cells, displaying a healthy nuclear envelope and a physiological state of chromatin condensation. In the cytoplasm compartment, a clearly defined rough endoplasmic reticulum (RER) and a normal mitochondrial morphology were observed.

Similarly, both young CP- and VP-treated fibroblasts displayed a healthy cellular organization, showing well-defined mitochondrial morphology characterized by the presence of clearly visible crests, a high amount of endoplasmic reticulum, and a normal Golgi apparatus.

**Figure 10:**
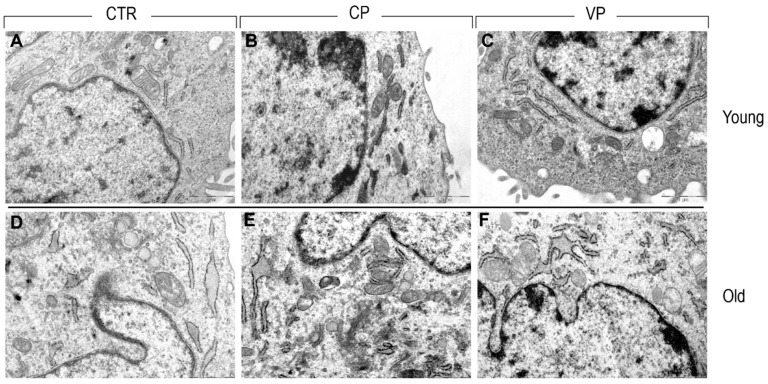
TEM ultrastructural analysis of ctr (**A**,**D**), CP (**B**,**E**) and VP (**C**,**F**) treatment, in both young and old NHDF (**A**–**C** and **D**–**F**, respectively).

In contrast, old fibroblasts exhibited a higher abundance of rough endoplasmic reticulum and an enlarged Golgi compared to young fibroblasts. Additionally, despite old NHDF experimental groups showing a well-preserved mitochondrial morphology, a reduction in the density of mitochondria was observed in the old control group compared to both Carmagnola and Voghera-treated fibroblasts. Moreover, while old treated (i.e., CP and VP NHDF) and untreated fibroblasts showed a healthy nuclear envelope in all aged experimental groups, fragmented RER was detected (Figure 10).

## 4. Discussion and Conclusions

Senescence is a complex event finely tuned by several environmental and endogenous mechanisms (e.g., oncogene activity, telomere uncapping, and oxidative stress) that can lead cells to exhibit specific characteristics, including irreversible growth arrest, altered protein expression, as well as enlarged and flattened morphology [25,26]. 

Recently, researchers have identified several compounds that can significantly reverse senescence by improving the above-mentioned senescence-related phenotype [27,28,29].

The great potential of ascorbic acid in preventing the natural aging process is well described in the literature, with particular attention to its involvement in maintaining the health and beauty of the skin [30,31,32]. Ascorbic acid has also been reported to extend replicative lifespan in human diploid fibroblast culture, possibly through a reduction in the rate of telomere shortening [33,34].

Based on our previous data demonstrating the antioxidant effect of Voghera pepper due to its high content of ascorbic acid, the purpose of the present study was to evaluate the possible anti-aging effect of Voghera pepper extract in young and old NHDF, focusing our attention on cell morphology, cellular ultrastructure, modulation of the cell cycle, and changes in the expression levels of (i) PCNA, a molecule involved in proliferation, DNA repair mechanisms, and cell cycle control and (ii) senescence markers (i.e., p16 and p53). 

Our present findings demonstrate that Voghera pepper extracts induce the recovery of a young-like morphology, S phase entry with consequent DNA synthesis during the cell cycle, and maintenance of healthy cellular ultrastructure in aged NHDF.

The tissue composition and proportion of fibroblast subpopulations play a crucial role in maintaining healthy connective tissue [35], as well as in disease [36]. Alteration of fibroblast morphology is one of the main features for the identification of a possible pathological condition, e.g., in giant cell fibroma, a type of tumor characterized by the presence of many enlarged stellate giant cells [37]. In the present study, old control NHDF observed by phase contrast microscopy showed an increase in cell area, which is approximately double compared with young control cells. However, Voghera pepper extract already allows young fibroblasts to maintain their physiological morphology and subsequently reduce the cytoplasmic area in old treated cells, stimulating aged fibroblasts to recover the young-like phenotype, unlike Carmagnola pepper-treated cells, where the cellular area increases even in young NHDF and seems unaffected in old CP-treated cells.

Additionally, the clonogenic assay results revealed that long-lasting (9 days) exposure to VP extracts induced colony formation stimulation in young NHDF without affecting the clonal capability of old NHDF, unlike CP extracts, which did not show a significant colony formation effect on young NHDF and even led to a reduction in clonal capability in old NHDF.

Although the reported alterations in the different cell cycle phases measured in differently treated NHDF could be attributable to biological variability, the slight increase in DNA synthesis highlighted by the increase in S-phase observed in both young and old NHDF after CP treatment, and the further increase observed in VP NDHF cells, could be linked to an improved proliferation activity due to the extract exposure related to the documented ability of ascorbic acid to promote cell entry into the S phase and its strong blocking effect on different mechanisms of cell death [38]. Cytofluorimetric analyses of living cells support this concept, as demonstrated by the increase in the number of live cells after pepper exposure in both young and old NDHF, corroborating the possible effects of pepper extract exposure on the maintenance of cell wellness in young fibroblasts and the inhibition of cell death mechanisms, promoting cell vitality also in aged treated cells.

The activation of p21 and p53 pathways plays a key role in regulating senescence, and prolonged overexpression of any of these components is sufficient to lead to senescence [39,40]. However, senescent fibroblasts that express low levels of p16 and show p53 downregulation can re-enter the cell cycle [25]. The p53 gene plays an important role in the regulation of cell division, and the increase in p53 expression levels in old cell lines observed in our results supports the idea of a senescence phenotype of old NHDF [41]. Moreover, based on these literature data, the reduced expression levels of p16 and the concomitant reduction in p53 activity observed after CP and VP exposure in our study could be linked to a potential block of the senescence process in young treated fibroblasts and a parallel reactivation of the cell cycle in old treated cells, resulting in a recovery of young-like phenotype in aged cells, as demonstrated by the similar expression levels of p16 and p53 between young control and old CP- or VP-treated fibroblasts. The reduced immunopositivity for p53 detected in treated cells corroborates the idea of a possible activation of the cell cycle, aiming to recover physiological cellular proliferation [42], in line with the observed increased S-phase measured in young and old treated NHDF. Although the dense diffuse nuclear p16 and p53 immunofluorescence detected in the nucleoplasm in our study is well documented in the literature as an unequivocal marker for the senescence process, the granular cytoplasmic immunoreactivity observed in both young and old untreated and differently treated NHDF suggests a possible involvement of the cellular localization of these senescent markers in several mechanisms, from the regulation of proliferation to the modulation of different regulated cell death mechanisms (e.g., apoptosis and autophagy), which could be finely regulated in young and old fibroblasts, to different extents, by our pepper extracts [43,44].

In parallel to this, to further strengthen the hypothesis of cellular activation of proliferative events after our phytotherapy treatment, although control young NHDF cells’ PCNA immunolabeling was homogeneously distributed in the nucleus, after treatment, several cells showed a spot-like fluorescence, most evident after VP, suggesting an active regulatory mechanism of the cell cycle and possible involvement of DNA repair mechanisms in these cells [45]. Furthermore, although PCNA expression levels are reduced with aging, as widely reported in the literature [45,46] and demonstrated in the present study, treatment with our extracts can lead to a reactivation of proliferation in aged fibroblasts, allowing old VP-treated cells to reach expression levels of PCNA comparable to those observed in the young control.

However, the data obtained using the clonogenic assay, after treating aged fibroblasts with pepper, show proliferation results apparently inconsistent with the expression levels of the proliferation marker PCNA. This divergent trend could be attributed to cellular interaction mechanisms. In fact, the analysis of clonogenic survival data based on plating efficiency is significantly influenced by cellular cooperation, leading to a substantial underestimation of intrinsic analysis errors in a considerable portion of cell lines [47].

Transmission electron microscopy allows us to underline the ultrastructural changes that occurred in our aged fibroblast models, demonstrating the presence of an enlarged Golgi apparatus, fragmented RER, and a reduction in mitochondria density in old NHDF. However, nuclear morphology seems to be unaffected by the aging process. Ultrastructural age-related changes have been extensively reported in the literature during the last century [48], such as the relationship between mitochondrial alteration and reactive oxygen species overproduction in the aging process [49,50,51]. Our pepper extracts seem to be able to increase the number of mitochondria in aged NHDF, suggesting a possible beneficial effect of pepper supplementation in the maintenance of functional and morphological integrity and consequently reducing the onset of the redox state in old NHDF.

The research on natural products in the fight against aging has been one of the most important topics in recent years in order to discover and develop new strategies to try to fight this physiological process, particularly in more industrialized countries, where life expectancy continues to advance, and the concept of healthy aging takes on a key role for the entire community. Ascorbic acid’s properties in regulating cell proliferation processes make this molecule a more than strong ally in the fight against aging, aiming to keep our cells young as long as possible. The high ascorbic acid content present in the Voghera pepper, two times higher than CP, makes this sweet bell pepper variety an important ally in slowing down the mechanisms involved in cellular aging, despite the total carotenoid values being slightly lower than those of the Carmagnola pepper. Therefore, this leads us to hypothesize that the observed anti-aging effects following treatment with these extracts are more plausibly attributed to the high concentration of ascorbic acid in the Voghera pepper, rather than its overall carotenoid content.

Bioactive compounds are molecules that typically occur in small quantities in food, especially in vegetables, and can be beneficial for health, having anticancer, anti-inflammatory, cytoprotective, neuroprotective, and anti-aging properties [52].

The mechanisms involved include not only free radical scavenging, but also activation of cytoprotective signal cascades, such as the Keap1–Nrf2–ARE pathway. This mechanism accelerates the expression levels of genes that regulate processes such as protein stability, autophagy, senescence, and protection against oxidative stress and inflammation. In this context, the biomolecules found in Voghera pepper extract might function as cytoprotective agents, reducing the occurrence of stressful conditions. This could subsequently lead to the inactivation of Keap1 and stabilization of Nrf2, ultimately resulting in the transcriptional activation of specific cytoprotective genes [53,54].

Thus, our future research will aim to quantify other lipid-soluble antioxidants (such as tocopherols) and water-soluble antioxidants like polyphenols and flavonoids, whose synergism may enhance cellular protection and prevent aging-related diseases.

The data obtained in this study demonstrate that Voghera pepper has a protective effect on aged NHDF, preserving the physiological cellular ultrastructure, reactivating cell proliferation mechanisms, and parallelly blocking the senescence events, leading to a partial recovery of young-like morphological and functional features in aged human fibroblast. Taken together, these findings support the idea that Voghera bell pepper supplementation could be a good natural strategy to prevent physiological aging, allowing healthy aging in humans, using local Italian vegetables with several documented beneficial effects for human health.

However, as a room for improvement, future research could benefit from using Ki67 or BrdU staining to further explore the extract’s potential to stimulate cell proliferation. Additionally, although p53 and p16 are useful markers for assessing the impact of our treatment on cellular senescence, the use of additional markers, such as beta-galactosidase, could offer a more comprehensive understanding of the extract’s effects on senescence mechanisms. Moreover, telomere shortening in our in vitro model could be evaluated to better investigate the aging process [55].

Additionally, both multinucleation and polyploidy (nuclear replication without nuclear division) have been reported in senescent fibroblasts [56,57], and one of the main features of multinucleated fibroblasts is the presence of enlarged cytoplasms [58]. Although we did not clearly detect polynucleated cells in our model, we plan to investigate this aspect further to better understand the senescence mechanism and cellular division process in the NHDF cell line.

Despite our natural extracts showing significant effects on our in vitro model already after a 24 h treatment, it would be valuable to examine the effects of pepper extract for longer (i.e., 48, 72, or 96 h) or continuous exposure over multiple passages. This extended investigation could reveal whether the extracts can mitigate aging and would better mimic a potential dietary intake of pepper supplements.

## Figures and Tables

**Table 1:** Summary description of preparation, features, and storage of extracts.

Composition	Fruit Body (i.e., Endocarp, Mesocarp and Pericarp)
Initial biomass	15 g
Extraction medium	water/1,2-propanediol (45–55%)
Stock solution concentration	150 mg/mL
Work solution concentration	1 mg/mL
Purification method	Membrane filtration
Storage	Dark environment and controlled temperature (−20 °C)
Ascorbic Acid (µg/g)	724.2 ± 0.37 (VP) 378.5 ± 0.25 (CP)
Total carotenoids (µg/g)	55.4 (VP) 112.5 (CP)

**Table 2:** Primary and secondary antibodies used for immunofluorescence reactions.

	Antigen	Immunogen	Manufacturer, Species, Mono-Polyclonal, Cat./Lot.	Dilution
Primary Antibodies	Anti-Proliferating Cell Nuclear Antigen (Ab-1)	Purified antibody raised against the ~37 kDa PCNA protein of mouse origin	Sigma-Aldrich (St. Louis, MO, USA), Mouse monoclonal IgG2a, Cat# NA03-200UG	1:500
	Anti-p53 (Ab-5)	Purified antibody raised against the ~53 kDa wild type p53 protein of mouse origin	Sigma-Aldrich (St. Louis, MO, USA), Mouse monoclonal IgG2a, Cat# OP33-100UG	1:100
	P16INK4a/CDKN2A	A synthetic peptide corresponding to a sequence within amino acids 20 to the C-terminus of human CDKN2A/p16INK4a	GeneTex (Irvine, CA, USA), Mouse monoclonal IgG, Cat# GTX03119	1:250
Secondary Antibodies	Alexa Fluor™ 594 goat anti-mouse IgG (H + L) Highly Cross-Adsorbed Secondary Antibody	Gamma immunoglobins Heavy and light chains	Thermo Fisher Scientific (Monza, Italy)	1:200

**Table 3:** Statistical analysis for cell area in young and old NHDF; * *p* < 0.05, ns: no significance. Data are expressed as the mean ± SEM.

	Y-CTR	Y-CP	Y-VP
Y-CTR (3240.21 ± 137.26)	--	*	ns
Y-CP (4281.35 ± 304.99)	*	--	*
Y-VP (3364.10 ± 258.55)	ns	*	--
	**O-CTR**	**O-CP**	**O-VP**
O-CTR (7911.64 ± 532.97)	--	ns	ns
O-CP (9487 ± 809.23)	ns	--	*
O-VP (6917.25 ± 631.65)	ns	*	--

**Table 4:** Statistical analysis for the clonogenic assay in young and old NHDF; ** *p* < 0.01, ns: no significance. Data are expressed as the mean ± SEM.

	Y-CTR	Y-CP	Y-VP
Y-CTR (100)	--	ns	**
Y-CP (118.37± 1.81)	ns	--	ns
Y-VP (149.66 ± 1.84)	**	ns	--
	**O-CTR**	**O-CP**	**O-VP**
O-CTR (100)	--	**	ns
O-CP (37.5 ± 2.62)	**	--	ns
O-VP (47.43 ± 2.81)	ns	ns	--

**Table 5:** Statistical analysis for PCNA expression in young and old NHDF; * *p* < 0.05, ns: no significance. Data are expressed as the mean ± SEM.

	Y-CTR	Y-CP	Y-VP
Y-CTR (67.65 ± 2.78)	--	ns	ns
Y-CP (59.16 ± 1.89)	Ns	--	ns
Y-VP (64.68 ± 3.08)	Ns	ns	--
	**O-CTR**	**O-CP**	**O-VP**
O-CTR (43.36 ± 4.67)	--	ns	*
O-CP (51.74 ± 3.77)	--	--	--
O-VP (57.69 ± 3.40)	*	--	--

**Table 6:** Statistical analysis for p53 expression in young and old NHDF; * *p* < 0.05, *** *p* < 0.001, ns: no significance.

	O-CTR	O-CP	O-VP
O-CTR (50.16 ± 3.41)	--	*	*
O-CP (18.10 ± 3.55)	*	--	ns
O-VP (8.40 ± 2.60)	*	ns	--
	**Y-CTR**	**Y-CP**	**Y-VP**
Y-CTR (13.56 ± 1.57)	--	***	ns
Y-CP (1.37 ± 0.32)	***	--	*
Y-VP (0.46 ± 0.25)	ns	*	--

**Table 7:** Statistical analysis for p16 expression in young and old NHDF; * *p* < 0.05, *** *p* < 0.001, ns: no significance. Data are expressed as the mean ± SEM.

	Y-CTR	Y-CP	Y-VP
Y-CTR (43.44 ± 2.68)	--	***	***
Y-CP (28.59 ± 2.45)	***	--	*
Y-VP (38.63 ± 2.93)	***	*	--
	**O-CTR**	**O-CP**	**O-VP**
O-CTR (61.87 ± 4.25)	--	***	***
O-CP (48.58 ± 4.05)	***	--	ns
O-VP (46.70 ± 3.79)	***	ns	--

## Data Availability

The data presented in this study are available in the article.
